# Epidemiological investigations of bovine besnoitiosis in Hungary

**DOI:** 10.1186/1756-3305-7-S1-O32

**Published:** 2014-04-01

**Authors:** S Hornok, W Basso, A Fedák, F Baska, L Dencső, T Abonyi, B Dénes

**Affiliations:** 1 Department of Parasitology and Zoology, Faculty of Vet. Science, SZIU, Budapest, Hungary; 2 Institute of Parasitology, Vetsuisse Faculty, University of Zürich, Switzerland; 3 Veterinary Authority, Miskolc, Hungary; 4 Department of Pathology, Faculty of Vet. Science, SZIU, Budapest, Hungary; 5 NÉBIH, Veterinary Diagnostic Directorate, Budapest, Hungary

## 

It usually gains proper attention, when blood-sucking arthropod species in Europe show northward spreading, in part due to climate change and global warming. Accordingly, it is well known, that the geographical regions where pathogens transmitted by these as biological vectors are endemic, also may extend towards the north. However, it is mostly less emphasized, that for vector-borne pathogens present in Western Europe the risk of eastward emergence is also possible, if they have competent vectors in the central and/or eastern part of our continent. *Besnoitia besnoiti *(Apicomplexa: Sarcocystidae) is a cyst-forming coccidian parasite that may cause severe lesions (with usually high seroprevalence, but low morbidity and mortality) in cattle as intermediate hosts. Wild ruminants are also susceptible. As a unique example among cystogenic coccidia, the main transmission route of *B. besnoiti *appears to be mechanical by blood-sucking dipterans (tabanid and stable flies), although it is also possible iatrogenically (with hypodermic needles) and most likely with close contact between animals. Bovine besnoitiosis has been endemic to South-Western Europe for more than a century, but a significant geographical expansion was observed during the last decade to other parts of the formerly endemic countries and to countries neighboring France.

During the autumn of 2013 bovine besnoitiosis was diagnosed in a beef cattle herd in Hungary (for the first time in Central-Eastern Europe), following the import of Aubrac heifers and bulls from France in the previous two years. The preliminary serological herd screening with ELISA shows that even after the 2nd year of the presence of imported animals (with high prevalence of *B. besnoiti *infection), seropositivity among local animals, which originally belonged to the herd, is low. Based on ELISA results venereal transmission (from imported, infected bulls to local, uninfected cows) appears to be either rare or unlikely. The seroprevalence of besnoitiosis decreased significantly among calves born to the group of imported mother cows. The risk of infection seems to be high, when calves stay with their mother during suckling (for 6-7 months), and if animals are kept in the same stable (although physically separated) during the main fly season. Confirmation of the ELISA results is done with immunoblot and IFAT. All seropositive cattle are now kept at a distance of several kilometres from other groups of animals, prior to culling. Molecular and sero-epidemiological evaluation of the situation continues with the aim of preventing the spread of the disease and regaining the epidemiological status of Hungary as exempt of bovine besnoitiosis.

